# Profiling symptom burden and its influencing factors at discharge for patients undergoing lung cancer surgery: a cross-sectional analysis

**DOI:** 10.1186/s13019-022-01974-9

**Published:** 2022-09-03

**Authors:** Jia Liao, Yaqin Wang, Wei Dai, Xing Wei, Hongfan Yu, Pu Yang, Tianpeng Xie, Qiang Li, Xiaoqin Liu, Qiuling Shi

**Affiliations:** 1 Department of Thoracic Surgery, Sichuan Cancer Hospital and Institute, Sichuan Cancer Center, School of Medicine, University of Electronic Science and Technology of China, Chengdu, Sichuan People’s Republic of China; 2 Center for Cancer Prevention Research, Sichuan Cancer Hospital, School of Medicine, University of Electronic Science and Technology of China, No. 55, Section 4, South Renmin Road, Chengdu, 610041 Sichuan People’s Republic of China; 3 School of Public Health, Chongqing Medical University, Chongqing, People’s Republic of China; 4 State Key Laboratory of Biomedical Engineering, College of Biomedical Engineering, Chongqing Medical University, Chongqing, People’s Republic of China

**Keywords:** Symptoms, Patient-reported outcomes, Lung cancer, Surgery, At discharge

## Abstract

**Background:**

Following lung cancer surgery, patients often experience severe symptoms which are not properly assessed at discharge. The aim of this study was to identify the clinical presentation at discharge and the influencing factors of postoperative symptoms in patients who have undergone lung cancer surgery.

**Methods:**

This cross-sectional study analysed data from patients who participated in a prospective cohort study that enrolled patients who underwent lung cancer surgery at six tertiary hospitals in the People’s Republic of China, from November 2017 to January 2020. Patient symptoms at discharge were measured using the MD Anderson Symptom Inventory Lung Cancer module. The five core symptoms were defined according to ratings of moderate to severe symptoms (≥ 4 on a 0–10 scale). A multivariate linear regression model was used to identify the influencing factors of each symptom at discharge.

**Results:**

Among the 366 participants, 51.9% were male and the mean (SD) age was 55.81 (10.43) years. At discharge, the core symptoms were cough (36.4%), pain (28.2%), disturbed sleep (26.3%), shortness of breath (25.8%), and fatigue (24.3%), and more than half of the participants (54.6%) had one to five of the core symptoms, with moderate to severe severity. A low annual income and the use of two chest tubes were significantly associated (*P* = 0.030 and 0.014, respectively) with higher mean scores of the core symptoms.

**Conclusion:**

Though clinically eligible for discharge, more than half of the participants had severe symptoms at discharge after lung cancer surgery. Special attention should be given to patients who have two chest tubes after surgery and those who have a low annual income.

## Background

Global cancer statistics for 2020 show that lung cancer has the highest mortality rate and is the most prevalent and the second most prevalent cancer in men and women, respectively [1]. In the People’s Republic of China, lung cancer remains the most common cancer type and is the leading cause of cancer-related deaths for both sexes, thereby accounting for 40% of global lung cancer-related deaths [2]. Surgery is considered the best curative option for operable lung cancer [3]. With minimally invasive video-assisted thoracoscopic surgery (VATS) for lung cancer, the patients’ length of hospital stay has decreased significantly [4]. Currently, most clinicians use clinical indicators to determine when to discharge a patient and these indicators do not include the patient's symptoms at discharge [5, 6]. However, physical healing should be an important determinant of recovery, and the return of mild symptoms is crucial because no clinical intervention is required and there is little impact on daily functioning, which thereby ensures that the patient can return to normal life [7].

Nonetheless, the patients’ perceptions of the severity or persistence of their symptoms are often overlooked in assessments that are based on clinical indicators. Patients who underwent surgery for lung cancer and were discharged with severe symptoms [8, 9] did not experience a return to baseline levels with regard to cough, pain, shortness of breath, sleep disturbance, and fatigue for 1–4 months after the surgery [10, 11, 12]. Moreover, there was a significant association between the reporting of severe symptoms, such as pain, cough, and shortness of breath, and readmission after discharge [13]. In addition, many patients with cancer require postoperative adjuvant therapy, such as chemotherapy, radiotherapy, targeted therapy, or a combination of these therapies [14]. Furthermore, persistent postoperative severe symptoms interfere with the functional recovery of patients and have a negative impact on their prognosis and timely return to their scheduled oncologic therapy [15]. Thus, patient symptom management is one of the most crucial care needs [9].

However, most of the previous studies on symptoms have focused on preoperative or post-discharge time-points, and little attention has been paid to the symptoms that are present at discharge [10, 16, 17]. Furthermore, in the context of VATS, the symptoms at discharge for patients after lung cancer surgery are unclear. Therefore, we conducted this study to identify the core symptoms at discharge and their influencing factors in patients who had undergone surgery for lung cancer.

## Methods

### Study design and participants

**Fig. 1:**
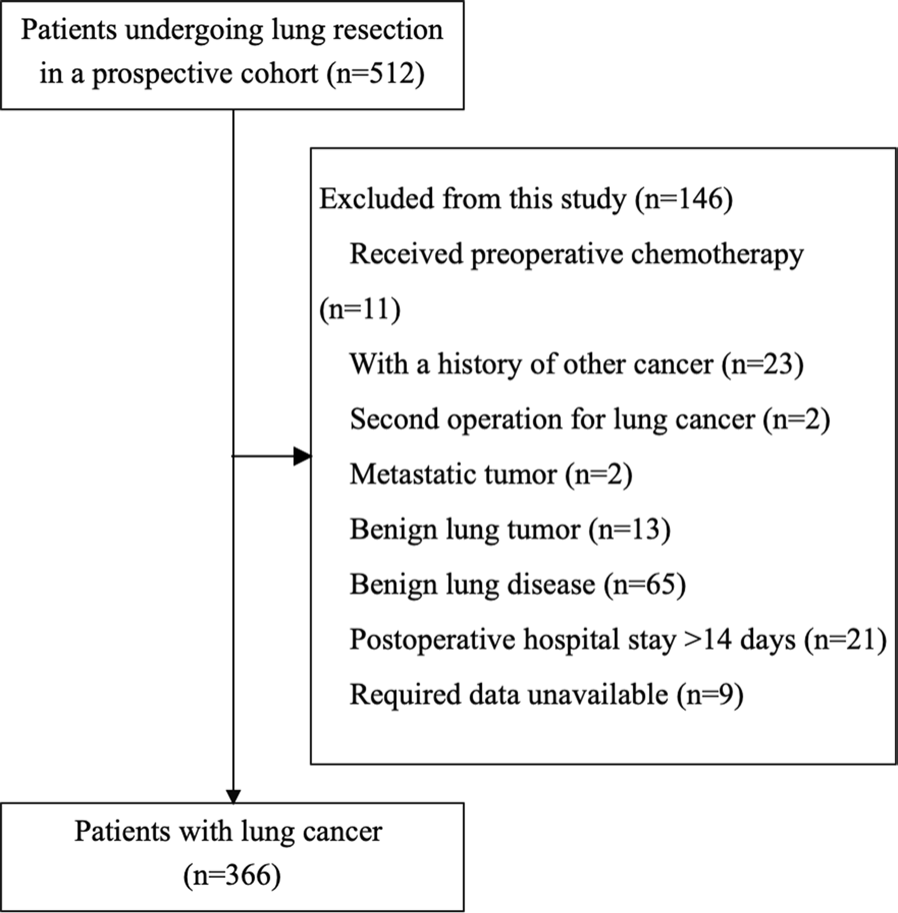
Participant selection flow diagram

This cross-sectional study involved an analysis of data that were extracted from a prospective, observational cohort study (CN-PRO-Lung 1, ClinicalTrials.gov identifier NCT03341377) [18], which included patients who underwent surgery for lung cancer at six tertiary hospitals in the People’s Republic of China from November 2017 to January 2020. This study was approved by the Ethics Committee of the Sichuan Cancer Hospital and by the respective ethics committees of the other study centres. All the participants provided written informed consent prior to their enrolment in the study. The inclusion criteria were: age ≥ 18 years, undergoing a lung resection, and a pathological diagnosis of primary lung cancer. The exclusion criteria were: a history of preoperative chemotherapy; history of other cancer, recurrence, or multiple primary lung cancer for the second operation; postoperative length of hospital stay > 14 days; and unavailability of the MD Anderson Symptom Inventory-Lung Cancer module (MDASL-LC) data recorded 1 day prior to or at discharge. Figure 1 shows the patient selection flow diagram.

### Data collection and outcome measures

Data on demographic and clinical variables, including age, sex, body mass index (BMI), operative time, highest level of education, annual income, smoking history, drinking history, American Society of Anesthesiologists (ASA) physical status classification, tumour pathological type and stage, postoperative maximum complication grade, Charlson Comorbidity Index (CCI), surgical approach, lymphadenectomy, and the number of chest tubes, were collected from the hospital’s electronic medical record system. The MDASI-LC [19] was used to measure the patient's symptoms and was recorded on paper or electronic devices. The patients were asked to ‘think back over the last 24 h and indicate the score’. The MDASI-LC was completed independently by the patient on the day before discharge or at discharge. The MDASI-LC includes 16 symptoms, and each symptom is rated on an 11-point scale, ranging from 0 (no symptoms) to 10 (severe symptoms). Based on the levels of pain intensity used in the National Comprehensive Cancer Network clinical practice guidelines for adult cancer pain, we defined pain intensity as mild (1–3 points), moderate (4–7 points), and severe (8–10 points) [20]. Patients without pain received a score of zero. In this study, the annual personal income was classified as low income (< 100,000 Renminbi [RMB]) and high income (≥ 100,000 RMB). Finally, all the data were entered into the Research Electronic Data Capture [21] platform that is hosted at Sichuan Cancer Hospital.

### Statistical analysis

Only valid data from both the preoperative and MDASI-LC questionnaires that were completed at discharge were included in the analysis in this study. The statistical analyses were performed using SPSS version 23.0 (SPSS, Inc.). Categorical variables are expressed as numbers (percentages) and continuous variables as means (standard deviations [SD]). We defined the five core symptoms based on the incidence and severity (moderate to severe). We used a multiple linear regression model to analyse the relationship between the demographic and clinical factors and the core symptoms. The mean score of the core symptoms was used as the dependent variable, and the demographic and clinical variables were used as the independent variables. In all the analyses, *p*-values < 0.05 were considered statistically significant.

## Results

### Participant characteristics

Of the 512 patients in the prospective cohort, 366 were eligible for inclusion in this study. The mean (SD) age of the patients was 55.81 (10.43) years; 51.9% were male, and 86.3% had an annual income < 100,000 RMB (Table 1). A substantial percentage of the participants had no history of smoking (63.9%) or drinking (75.4%), had an ASA Physical Status Classification Score ≥ II (54.6%), and had a CCI score ≥ 1 (68.6%). Most of the participants had an adenocarcinoma (83.6%), that was detected at an early stage (65.3%), and had no or Grade I postoperative complications (83.9%). Most of the participants received VATS (82.2%), a non-systematic lymph node dissection (60.1%), and had one chest tube inserted (69.4%).

**Table 1:** Demographics and clinical characteristics of patients

Variables	Mean ± SD or n (%)
Age, years	55.81 ± 10.43
BMI, kg/m^2^	22.99 ± 2.80
Operative time, minutes	136.77 ± 50.99
Sex	
Male	190 (51.9)
Female	176 (48.1)
Highest level of education	
Middle school and below	174(47.5)
Above middle school	192(52.5)
Annual income	
Low annual income (< 100,000 RMB)	316(86.3)
Hing annual income (≥ 100,000 RMB)	50(13.7)
Smoking history	
No	234 (63.9)
Yes	132(36.1)
Drinking history	
No	276(75.4)
Yes	90(24.6)
ASA physical status classification	
I	166(45.4)
≥ II	200(54.6)
Tumour pathological type	
Adenocarcinoma	306(83.6)
Non-adenocarcinoma	60(16.4)
Tumour pathological stage	
Early stage (0–I)	239(65.3)
Advanced stage (II–IV)	127(34.7)
Postoperative maximum complication grade	
None or I	307(83.9)
≥ II	59(16.1)
Charlson Comorbidity Index	
0	115(31.4)
1–5	251(68.6)
Surgical approach	
Video-assisted thoracoscopic surgery	301(82.2)
Open surgery	65(17.8)
Lymphadenectomy	
Systematic lymph node dissection	146(39.9)
Non-systematic lymph node dissection	220(60.1)
Number of chest tubes	
One	254(69.4)
Two	112(30.6)

*BMI* body mass index, *SD* standard deviation, *ASA* American Society of Anesthesiologists

### Symptoms at discharge

The prevalence and severity of the symptoms at discharge are presented in Table 2. In the order of prevalence (score ≥ 1 on a 0–10 scale), cough (92.3%), pain (90.7%), shortness of breath (81.4%), fatigue (79.5%), and disturbed sleep (72.1%) constituted the core symptoms. The five core symptoms that ranged from moderate to severe (score ≥ 4 on a 0 to 10 scale) were cough (36.4%), pain (28.2%), shortness of breath (25.8%), fatigue (24.3%), and disturbed sleep (26.3%).

**Table 2:** Overall symptom burden of 366 patients at discharge

Items	Available cases	Prevalence (rated ≥ 1), n (%)	Moderate to severe (rated ≥ 4), n (%)	Interquartile range		
				P_25_	P_50_	P_75_
Coughing	365	337 (92.3)	133 (36.4)	1	3	4
Pain	365	331 (90.7)	103 (28.2)	1	2	4
Shortness of breath	365	297 (81.4)	94 (25.8)	1	2	4
Disturbed sleep	365	263 (72.1)	96 (26.3)	0	2	4
Fatigue	366	291 (79.5)	89 (24.3)	1	2	3
Dry mouth	366	273 (74.6)	70 (19.1)	0	1	3
Lack of appetite	365	253 (69.3)	64 (17.5)	0	1	3
Drowsiness	366	253 (69.1)	71 (19.4)	0	1	3
Constipation	366	226 (61.7)	75 (20.5)	0	1	3
Distress	365	219 (60)	72 (19.7)	0	1	3
Sore throat	366	216 (59.0)	61 (16.7)	0	1	2
Difficulty remembering	365	213 (58.4)	60 (16.4)	0	1	2
Sadness	366	188 (51.4)	57 (15.6)	0	1	2
Numbness or tingling	366	151 (41.3)	30 (8.2)	0	0	2
Nausea	362	132 (36.5)	20 (5.5)	0	0	1
Vomiting	364	96 (26.4)	14 (3.8)	0	0	1

At discharge, more than half of the participants (54.6%) had one to five of the moderate-to-severe intensity common five symptoms, with 19.67% having one moderate to severe symptom, as well as 9.28%, 7.38%, 10.66%, and 7.65% having two, three, four, and five symptoms, respectively (Table 3).

**Table 3:** The number of any top 5 symptoms with scores ≥ 4 at discharge

Items	Number of patients	Percentage
No symptoms	166	45.36
One symptom	72	19.67
Two symptoms	34	9.28
Three symptoms	27	7.38
Four symptoms	39	10.66
Five symptoms	28	7.65

Top 5 symptoms include coughing, pain, shortness of breath, disturbed sleep, and fatigue

### Factors affecting the five core symptoms

Table 4 shows that participants with a low annual income (partial regression coefficient = ** − **0.626, *P* = 0.030) and with two chest tubes (partial regression coefficient = 0.515, *P* = 0.014) had more severe symptoms at discharge. However, the other variables did not show a statistically significant association with more severe symptoms at discharge.

**Table 4:** Multivariate linear regression analysis of factors that affected the mean score of the top 5 symptoms

Variables	Partial regression coefficient	SE	*P*-value
Age, years	0.003	0.014	0.809
BMI, kg/m^2^	− 0.006	0.034	0.856
Operative time, minutes	7.925	0.002	0.968
Sex	0.063	0.254	0.806
Male			
Female			
Highest level of education	− 0.003	0.197	0.987
Middle school and below			
Above middle school			
Annual income	− 0.626	0.287	0.030
Low annual income (< 100,000 RMB)			
High annual income (≥ 100,000 RMB)			
Smoking history	− 0.396	0.278	0.155
No			
Yes			
Drinking history	0.219	0.260	0.399
No			
Yes			
ASA physical status classification	0.354	0.196	0.071
I			
≥ II			
Charlson Comorbidity Index	0.049	0.298	0.869
0			
1–5			
Tumour pathological type	0.141	0.294	0.631
Adenocarcinoma			
Others			
Tumour pathological stage	0.348	0.233	0.137
Early stage (0–I)			
Advanced stage (II–IV)			
Postoperative maximum complication grade	− 0.433	0.254	0.089
< II			
≥ II			
Surgical approach	− 0.197	0.293	0.501
Video-assisted thoracoscopic surgery			
Open surgery			
Lymphadenectomy	− 0.378	0.374	0.313
Systematic lymph node dissection			
Non-systematic lymph node dissection			
Number of chest tube	0.515	0.209	0.014
One			
Two			

*BMI* body mass index, *ASA* American Society of Anesthesiologists, *SE* standard error

## Discussion

The results of our study showed that the core symptoms at discharge were cough, pain, disturbed sleep, shortness of breath, and fatigue. Though the patients were clinically eligible for discharge after undergoing lung cancer surgery, more than half of the participants experienced one or more of the core symptoms at the time of discharge. The severity of these symptoms ranged from moderate to severe. The incidence of these core symptoms was higher in patients with a low annual income or in those who had two chest tubes after surgery.

Our study showed that cough was the most prevalent of all symptoms at discharge, followed by pain, shortness of breath, fatigue, and disturbed sleep. Similarly, a previous study showed a prevalence of 25–50% [22, 23] of cough in discharged patients. Lung surgery inevitably results in trauma and anatomical alterations, such as lymph node dissection, bronchial sutures, diaphragmatic elevation, unilateral lung volume loss, and residual lobe deformation, all of which induce persistent postoperative cough [24]. The degree and duration of postoperative cough can affect the post-discharge quality of life of the patient [25]. Up to 69% of patients with lung cancer experience moderate to severe postoperative pain, and persistent postoperative pain may interfere with postoperative recovery and affect the development of complications [16]. Our study showed a lower percentage (28.2%) of moderate to severe postoperative pain, which could be related to the high proportion of VATS [26, 27]. In our study, the incidence of shortness of breath at discharge was 25.8%, which when severe was generally considered a reason for readmission [13]. Furthermore, fatigue has been reported as one of the most common and severe symptoms at each time point after thoracotomy-based surgery [28] and has a negative impact on the patient’s ability to receive postoperative cancer treatment as well as the patient’s long-term prognosis [29]. In this study, the prevalence of moderate to severe sleep disturbances was 26.3%, which has been reported as the most common symptom in surgical patients [30]. Compared to other reports, the differences between the patient-reported core symptoms and their lower scores in this study may be related to the use of different patient-reported outcome-measurement instruments and the different time points of outcome measurement.

In this study, we found that more than 50% of the participants reported the presence of one or more of the core symptoms whereas more than 25% of the participants reported three or more core symptoms at the time of discharge. Though most of the existing clinical studies have focused only on one symptom, patients rarely present with a single symptom, but instead with multiple symptoms that occur simultaneously [30] and may or may not be related to each other [31]. In the study by Trine et al., symptoms in patients who underwent lung cancer surgery often occurred in clusters and showed strong interrelationships [32], and the occurrence of symptom clusters was closely related to the patient’s quality of life [33]. Future studies of symptom management should focus on the assessment of the relationship between multiple symptoms, specific interventions, and patient outcomes [31].

Studies have shown that patients with cancer who have lower annual incomes are more likely to have severe symptoms [34, 35]. Our analysis showed that the socioeconomic status of patients with lung cancer was one of the factors that are related to symptom severity. This symptom is related to a lack of access to proper care, poor social support, and increased financial stress [35]. The medical cost of cancer treatment imposes a heavy burden on society and on the patients’ families. Furthermore, patients who experience economic pressure have more severe symptoms and a poorer quality of life [35]. In addition, the number of chest tubes that are inserted is a factor that contributes to the development of core symptoms. We found that patients with two chest tubes had more severe symptoms. Moreover, previous studies have reported significantly less pain in patients with a single chest tube after surgery [36, 37]. Thus, special care management strategies should be developed for patients with two or more chest tubes to reduce their burden of symptoms. Some studies [38, 39] that compared patients with different surgical approaches showed differences in the severity of the symptoms [40], but these findings differ from the results of our study. A reason for this difference may be that, instead of a single symptom, the mean score of the core symptoms in this study was used as the dependent variable. In addition, data were collected on the day of discharge, rather than during the postoperative period in this study. In an era of widespread use of VATS, a focus on symptoms at discharge and the factors that influence these symptoms will help establish clinically actionable post-discharge patient management strategies.

This study had some limitations. First, the study included only the annual income of the patient and not of their families. Personal incomes are imperfect measures of socioeconomic status as they may not reflect the household’s financial status [35]. Future studies will need to include more details to analyse the relationship between the household economic income and the patients' burden of symptoms. Second, though the instrument used to assess patient-reported outcomes in this study was the MDASI-LC, which is one of the four international lung cancer-specific instruments that has been verified and validated in regional populations [41], the validation study for the MDASI-LC was conducted in patients who were undergoing chemo-radiotherapy [18]. Thus, the MDASI-LC may not constitute the best instrument for assessing patient-reported outcomes in patients who were undergoing surgery. Third, our study was not free of bias. Patients with poor literacy skills did not participate in the study, and this limitation may have affect the generalizability of the conclusions of this study.

## Conclusions

Though clinically eligible for discharge, more than half of the participants in this study reported moderate-to-severe core symptoms at discharge. These core symptoms were significantly associated with a low annual income and the use of two chest tubes. For better patient recovery, we need to reconsider symptom-management strategies before the patient is discharged from the hospital.

## Data Availability

The datasets used and/or analysed during the current study are available from the corresponding author on reasonable request.

## Abbreviations

VATS: Video-assisted thoracoscopic surgery
MDASI-LC: MD Anderson Symptom Inventory-Lung Cancer
ASA: American Society of Anesthesiologists
